# Precision of coaxial needle placement in computed tomography-guided transthoracic needle biopsy

**DOI:** 10.3892/etm.2013.1283

**Published:** 2013-09-02

**Authors:** RUI HUANG, NAN-CHUAN JIANG, HAO-HAO LU, YU-HUI WANG, HUI LI, HE-SHUI SHI, PING HAN

**Affiliations:** Department of Radiology, Union Hospital, Tongji Medical College, Huazhong University of Science and Technology, Wuhan, Hubei 430022, P.R. China

**Keywords:** biopsy, computed tomography, thorax, audit

## Abstract

In the present study, a set of self-designed measurement protocols for the precision of coaxial needle placement (PCNP) was proposed and applied in a computed tomography (CT)-guided transthoracic needle biopsy (TNB) audit of an interventional radiologist to determine if the PCNP was commensurate with the experience of the operator. A total of 102 patients (98 with lung lesions and four with mediastinum lesions) consented to be subjected to CT-guided TNB performed by staff interventional radiologists. The patients were divided into two groups based on appointment date. Group A consisted of the first 51 patients and group B comprised of the latter 51 patients. A set of self-designed measurement protocols for PCNP was proposed, and the PCNP was classified into four grades, from grade 1 (most accurate) to grade 4 (least accurate). PCNPs were independently measured by three staff radiologists who were blind to the grouping. The anatomical features of the lesions were also analyzed between the two groups. A significant difference in the PCNP gained after the first needle placement was identified between the two groups (P=0.003, two-tailed). The number of patients in group B with grade I PCNP (51.0%) was significantly higher than that in group A (21.6%) (P<0.05). The number of patients in group B with grade III PCNP (11.8%) was significantly lower than that in group A (29.4%, P<0.05). The PCNP was observed to be commensurate with the experience of the operator and should be considered as a routine audit index in CT-guided TNB.

## Introduction

Computed tomography (CT)-guided transthoracic needle biopsy (TNB) has become a well-established procedure. It has been reported that CT-guided TNB is a relatively safe and accurate method of diagnosing suspected thoracic lesions (1–4). The general procedure of CT-guided TNB is as follows (3–10): With CT guidance, a coaxial needle (consisting of an outer cannula and an introducer stylet used for positioning the outer cannula) is inserted through the skin until it reaches the thoracic lesion. The introducer stylet is then removed while keeping the outer cannula in position. A biopsy gun or needle is then passed through the outer cannula for the sampling procedure. The precise insertion of the coaxial needle into the target lesion is a crucial step for avoiding injuries to important adjacent structures during CT-guided TNB. The precise insertion requires the operator to be exceptionally skillful. In 2003, the British Thoracic Society recommended that operators retrospectively audit their own practice (11). Tsai *et al* (5) further suggested a post-procedural review of biopsy cases in order to check how closely the procedure followed the plan of the operator. There are many factors involved in CT-guided TNB auditing, including monitoring the adequacy of biopsies, complications, pathological results and how closely the procedure follows the plan of the operator (i.e., the precision of coaxial needle placement, PCNP). However, to the best of our knowledge, no index for assessing the PCNP has been reported.

In the present study, a set of self-designed measurement protocols for PCNP were proposed and applied in a CT-guided TNB audit of an interventional radiologist to determine whether the PCNP was commensurate with the experience of the operator.

## Materials and methods

### Concepts

#### Biopsy area

Prior to coaxial needle placement, chest CT images were carefully reviewed in order to select an appropriate biopsy area that avoided the necrotic region.

#### Most appropriate route

The most appropriate route of needle insertion was determined prior to coaxial needle placement in order to lower the probability of complications and to ensure that tissue sampling was successful. An appropriate route leads towards the biopsy area while avoiding bones, adjacent vital organs, large vessels or bronchi, bullae and emphysematous areas.

#### Predefined sampling position (PSP)

The PSP is the target position of the coaxial needle tip at the end of the most appropriate route. The PSP was identified in a transverse CT image (slice thickness, 1–2 mm) prior to coaxial needle placement. For patients with lesions close to or adjoining a pleura, the distance between the PSP and the visceral pleura should not be <0.5 cm. This separation ensures that the occurrence of pneumothorax is avoided following withdrawal of the introducer stylet. The PSP is identified using anatomical features, including spiculation and lobulation of a lesion edge, and calcification and cavitation within a lesion and in adjacent bronchi or vessels.

#### Repeat coaxial needle placement (RCNP)

When the position of the coaxial needle tip was incorrect, the needle was withdrawn from the thoracic wall and then inserted again to the same PSP.

#### PCNP measurement protocols

**Figure 1.**
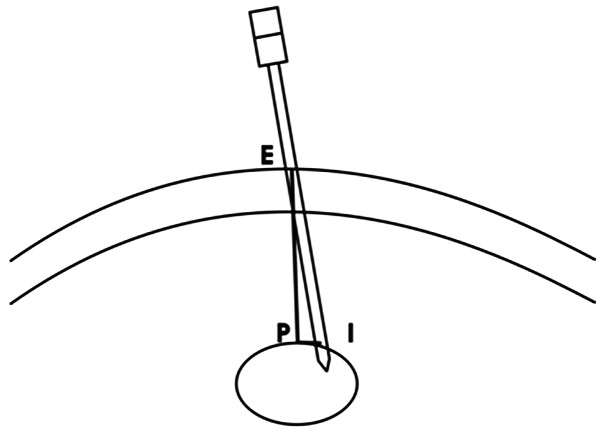
Diagram of the PCNP measurement protocol. E, skin entry site; P, predefined sampling position (PSP); EP, the most appropriate route. EI is the line in which the coaxial needle lies. PI is the line perpendicular to EP. I is the intersection of PI and EI. PCNP, precise coxial needle placement.

The CT images of the coaxial needle placement were transferred to a Siemens workstation (Leonardo; Siemens Medical Solutions, Erlangen, Germany). A multiplanar reformation (MPR) software environment was used. When the left button on the corresponding position of the PSP in the transverse CT image was clicked, the origin of the coordinates automatically shifted to the PSP position. The plane (Fig. 1) containing the tip of the coaxial needle, the skin entry site and the origin of coordinates (i.e., PSP) were defined by carefully rotating the three axes while keeping the origin of the coordinates in position. The PSP depth is the length of the line segment EP, the coaxial needle deviation from PSP is the length of the line segment PI (perpendicular to line segment EP) and the PCNP is the ratio of the coaxial needle deviation from the PSP to the PSP depth (i.e., PI/EP). The PCNP was classified into four grades as follows: grade I, PCNP<0.1; grade II, 0.1≤PCNP<0.2; grade III, 0.2≤PCNP<0.3; and grade IV, PCNP≥0.3.

#### Subjects

Between January 2009 and December 2010, a staff interventional radiologist (R.H.) was designated to carry out the CT-guided TNB procedures in Union Hospital, Tongji Medical College, Huazhong University of Science and Technology (Wuhan, China). During this period, a total of 102 patients (98 with lung lesions and four with mediastinum lesions) agreed to receive a CT-guided TNB. The indication for the procedure was determined by the physician who referred the patient and the interventional radiologist was required to agree with the procedure. The records of these patients were extracted from the institutional electronic archives and divided into two groups based on appointment date. Group A consisted of the first 51 patients (33 male and 18 female; mean age, 54.0 years; age range, 19–81 years), whereas group B comprised the latter 51 patients (37 male and 14 female; mean age, 51.5 years; age range, 17–73 years). This study was conducted in accordance with the Declaration of Helsinki and with approval from the Ethics Committee of Tongji Medical College, Huazhong University of Science and Technology. Written informed consent was obtained from all participants prior to CT-guided TNB.

#### Guiding equipment

All procedures were performed under the guidance of two multislice computed tomography (MSCT) imagers (Sensation 16 and Somatom Definition AS+; Siemens Medical Solutions). Selection of the guiding equipment was made according to routine arrangements. The scan parameters were 100 or 120 kV; 25–180 mAsec (CARE Dose or CARE Dose 4D technique); kernel B35 or B50; 512×512 matrix; 16×0.75 mm or 128×0.6 mm collimation; 1.0–2.0 mm slice thickness; 1.2 pitch; and a lung window setting (center = −400, width = 1400) or a mediastinum window setting (center = 0, width = 400).

#### Pre-procedural preparation

A coagulation screening, which consists of prothrombin time, activated partial thromboplastin time and platelet count, was performed before all procedures. Any anticoagulants or platelet inhibitors, such as aspirin, were withheld. All available thoracic images were reviewed by the operator prior to the biopsy procedure. The patients were instructed to refrain from moving, coughing or breathing deeply during the procedure. The importance of the patient holding their breath following the instructions of the operator at the end of a normal inhalation was explained to each patient. The technique was practiced before the procedure until the operator was satisfied.

#### Biopsy procedures

The patients fasted for at least 4 h prior to the procedure. The procedure was performed with the patient in a prone, supine or lateral decubitus position, depending on the location of the lesion. The biopsy procedure was standardized. A skin marker (a radiopaque thin stick or grid) was placed on the body surface corresponding to the target lesion. Preliminary unenhanced MSCT images (slice thickness, 1–2 mm) were obtained through the thoracic lesion when the patient held their breath at the end of a normal inhalation. The biopsy area and the most appropriate route, which included the skin entry site and PSP, were identified in the transverse CT images prior to coaxial needle placement. The patient was advanced into the gantry again and stopped at the skin entry site. The selected skin entry site was marked using the skin marker and the integrated laser beam. The skin entry site was then prepared and draped in a sterile manner. A local anesthetic was injected through the skin entry site into the level of the parietal pleura. The anesthetic needle was kept in an extrapulmonary position and a CT-scan was then performed to verify if the position of the skin entry site was correct. The anesthetic needle was then replaced with a 17-gauge coaxial needle (TruGuide; Bard Biopsy Systems, Tempe, Arizona, USA). In between breaths held by the patient at the end of a normal inhalation phase, the coaxial needle was gradually advanced to the PSP. The needle position was checked several times during the procedure by using intermittent CT guidance (slice thickness, 1–2 mm). If the position of the coaxial needle tip was confirmed to be correct, a sampling of the lesion was performed at least once using a matching 18-gauge biopsy gun (Max-Core, Bard Biopsy Systems). If the position of the coaxial needle tip was confirmed to be incorrect even after manipulation, an RCNP was then performed. The specimens were fixed in 10% formalin for pathological evaluation. Sections of the specimens were placed in a normal saline solution for microbiological examination to check for inflammatory disease. The procedure was successfully carried out in all cases.

#### Post-procedure imaging and care

Approximately 10 min after the coaxial needle was withdrawn, an unenhanced MSCT scan (slice thickness, 10 mm) was performed on the whole thorax to check for immediate pneumothorax and hemorrhage. The patients were placed in a puncture-side-down position and then monitored in a respiratory ward for at least 24 h. If a small, asymptomatic, immediate pneumothorax developed, the patient was conservatively treated by the administration of supplemental oxygen. A follow-up chest radiography was performed to evaluate the stability of the pneumothorax. Patients whose pneumothorax was worsening or was accompanied by symptoms, such as respiratory distress, shortness of breath, pain and decreased oxygen saturation, received chest tube insertions. Patients with hemoptysis, lung hemorrhage and hemothorax received hemostasis. Patients without complications or with stable mild complications were discharged after 24 h of observation. The discharged patients were instructed to return to the nearest emergency department if symptoms, including substantial pain and shortness of breath, developed following dismissal.

#### Data collection and statistical analyses

Following biopsy, all CT images that included information on thoracic lesions, skin entry site, PSP, actual needle trajectory, RCNP and post-procedural thorax CT scan were immediately recorded on a compact disc. Thoracic lesion reviews and PCNP measurements were performed by three staff radiologists (N.C.J., H.H.L. and Y.H.W. who had seven, four and three years experience, respectively). Data concerning lesion location (defined as the location of the lesion center), size (defined as the maximal axial diameter of the lesion), shape (nodules or tumors, exudation or consolidation, cavity) and PSP depth (distance from the skin entry site to PSP) were collected. The PCNP measurement and the PCNP grading gained after the first needle placement were determined according to the previously described protocols. Lesion location and shape were reviewed by the three radiologists and any differences of opinion were resolved by consensus. The lesion size, PSP depth and PCNP were independently measured by the three radiologists without knowledge of the groupings and results.

A thoracic lesion comparison between the two groups was performed using a Pearson’s Chi-square test. The PCNPs of groups A and B were compared using a two-tailed Student’s t-test. The first PCNP grading comparison between the two groups was performed using a Pearson’s Chi-square test. These tests were performed using the Statistical Product and Service Solutions software (version 16.0, SPSS, Inc., Chicago, IL, USA). P<0.05 was considered to indicate a statistically significant difference.

## Results

The comparison of the thoracic lesions between the two groups is summarized in Table I. No significant differences were identified in the lesion location, size or shape. The number of patients with a PSP depth of <4 cm was significantly smaller in group B than in group A (P=0.426).

The first PCNP was 0.19±0.12 in group A and 0.12±0.10 in group B. The difference in the first PCNP between the two groups was identified to be statistically significant (P=0.003, two-tailed). The comparison of the first PCNP grading between the two groups is summarized in Table II. The number of patients with grade I PCNP in group B was significantly higher than that in group A (P<0.05). The number of patients with grade III PCNP in group B was significantly lower than that in group A (P<0.05).

In group A, five patients underwent one RCNP. An improvement in the PNCP was observed in all five patients; however, only four patients had an increase in the PCNP grade (two patients from grade II to I, one patient from grade III to I and one patient from grade IV to III) and one patient remained in the same PCNP grade. In group B, three patients underwent one RCNP. An improvement in the PNCP was observed in all three patients, but only two patients had an increase in the PCNP grade (one patient from grade III to I and one patient from grade II to I) and one patient remained in the same PCNP grade. A patient in group B underwent four RCNPs (the patient had a target pulmonary nodule that had a diameter of 0.7 cm in the right lower lobe); although the first PCNP grade was I, the nodule was not reached until the fourth RCNP was performed.

## Discussion

The precise placement of the coaxial needle in the target lesion area is a crucial step in CT-guided TNB for a number of reasons. Firstly, given that CT screening is widely used for lung cancer (12–15), it is important to analyze unidentified small pulmonary nodules found at an early stage. This analysis is usually done by sampling via precise placement of the coaxial needle. Secondly, large pulmonary neoplasms often have necrotic areas. The cellular components of specimens obtained in these areas have little diagnostic significance; therefore, the use of these specimens is not suitable for cytological and pathological evaluations (16). In the present study, the operator carefully studied the CT images of the target lesions and then predetermined the biopsy area suitable for sampling prior to each CT-guided TNB. Thirdly, the biopsy procedure may injure pleura, adjacent vital organs, large vessels and bronchi, and may cause complications, including pneumothorax, hemothorax, hemoptysis, lung hemorrhage and air embolism (1,2,17–20). On rare occasions, the procedure may even result in life-threatening complications (18–20). The precise placement of the coaxial needle helps to minimize damage and complications.

PCNP measurement is difficult to evaluate due to multiple affecting factors, including the lesion size and depth, the experience of the operator, guiding techniques, the choice of puncture technique, the type of needle used and the level of cooperation of the patient. Among these factors, the guiding techniques, choice of puncture technique and type of needle used are stable factors in a number of institutes. The maximum cooperation of a patient may also be achieved via a preprocedural drill. Therefore, the lesion size and location and the experience of the operator are considered as the main factors that affect the PCNP measurement. According to numerous operators, the coaxial needle placement is more difficult when the thoracic lesion is deeper or smaller. The PCNP measurement protocols in this study minimize the effects of thoracic lesion size and depth during coaxial needle placement. By defining the PCNP as the ratio of the coaxial needle deviation to the PSP depth, the impact of lesion depth was reduced to a minimum. By defining the PSP at the end of the most appropriate route as the target point of the coaxial needle placement, the impact of lesion size was also significantly decreased. The three radiologists involved in this study all stated that the protocols were easy to follow.

The results demonstrated that a better PCNP was obtained in group B than in group A. This indicates that the PCNP increases as the experience of the operator increases. The results were consistent with our expectations.

This set of protocols has mainly been used for assessing the PCNP of the operator. However, these PCNP measurement protocols are intended to have further potential uses. New technologies and methods of CT-guided TNBs are emerging. Thus, the PCNP measurement protocols may be used to assess objectively if these new technologies and methods are able to help to improve the PCNP of the operator.

Although a Siemens image workstation and MPR software were used in this study, any image workstation or MPR software would fulfill the demand of the protocols as long as the origin of the coordinates remains in position while the three axes are rotated.

The PCNP measurement protocols do have limitations. For example, the PSP is identified using anatomical features and inaccuracies are inevitable. The optimal method of avoiding inaccuracies is for the operator to study the CT images carefully in advance.

## Figures and Tables

**Table I.** Comparison of thoracic lesions between two groups.

Variable	Group A	Group B	P-value^a^
Lesion location			
Right upper lobe	13 (25.5)	15 (29.4)	0.657
Right middle lobe	4 (7.8)	4 (7.8)	1.000^b^
Right lower lobe	8 (15.7)	11 (21.6)	0.445
Left upper lobe	13 (25.5)	12 (23.5)	0.818
Left lower lobe	11 (21.6)	7 (13.7)	0.299
Mediastinum	2 (3.9)	2 (3.9)	1.000^b^
Lesion size			
<3 cm	10 (19.6)	8 (15.7)	0.603
3–6 cm	22 (43.1)	20 (39.2)	0.687
>6 cm	19 (37.3)	23 (45.1)	0.421
Lesion shape			
Nodules or tumors	43 (84.3)	44 (86.3)	0.78
Exudation or consolidation	5 (9.8)	2 (3.9)	0.433^b^
Cavity	3 (5.9)	5 (9.8)	0.713^b^
PSP depth			
<4 cm	25 (49.0)	15 (29.4)	0.043
4–6 cm	21 (41.2)	25 (49.0)	0.426
>6 cm	5 (9.8)	11 (21.6)	0.102

a P-values generated by Chi-square tests for categorical variables;

b Continuity correction was performed to determine significant difference. Data are numbers of patients; data in parentheses are percentages. PSP, predefined sampling position.

**Table II.** Comparison of the first PCNP grading between the two groups.

PCNP grading	Group A	Group B	P-value^a^
I	11 (21.6)	26 (51.0)	0.002
II	19 (37.3)	15 (29.4)	0.401
III	15 (29.4)	6 (11.8)	0.028
IV	6 (11.8)	4 (7.8)	0.505

a P-values generated by Chi-square tests for categorical variables. Data are numbers of patients; data in parentheses are percentages. PCNP, precise coaxial needle placement.
